# The contribution of miR-122 to the innate immunity by regulating toll-like receptor 4 in hepatoma cells

**DOI:** 10.1186/s12876-019-1048-3

**Published:** 2019-07-24

**Authors:** Liyu Shi, Xiaoqiu Zheng, Yuzhuo Fan, Xiaolan Yang, Aimei Li, Jun Qian

**Affiliations:** 1 Department of Microbiology, Harbin Medical University, No. 194, Xuefu Road, Harbin, 150081 Heilongjiang province China; 2 Wu Lien-Teh institutes, Harbin Medical University, Harbin, Heilongjiang province China

**Keywords:** MiRNA, miR-122, Toll like receptor, Hepatocytes

## Abstract

**Background:**

Hepatocellular carcinoma (HCC) is a kind of malignancies to impact human health. It has been reported that aberrant toll-like receptor (TLR) signaling may contribute to the development and progression of HCC, especially TLR4. MiR-122, which extensively involved in hepatitis virus infection and the apoptosis of hepatoma cells, might be decreased in HCC patients livers. The hypothesis of this study was whether miR-122 plays a role in inflammatory pathways through regulating TLR4 expression in hepatoma cells.

**Methods:**

The expression of miR-122 in the tissues of HCC patients compared to controls in TCGA datasets was analyzed. The relationship between miR-122 and TLR4 was detected in HCC cell lines by increasing/decreasing miR-122 expression. The target of miR-122 on TLR4 was confirmed by luciferase reporter assays. The proliferation of HCC cells and production of proinflammatory cytokines were measured with miR-122 upregulation and inhibition.

**Results:**

We found that the expression of miR-122 was decreased in HCC tissues and showed the diagnostic capacity for HCC in TCGA datasets. MiR-122 and TLR4 expression have negative correlation in normal liver cells and HCC cells. Upregulation of miR-122 significantly inhibited TLR4 expression in hepatoma cells, including in hepatoma cells with the induction of LPS, while knocking down miR-122 increased TLR4 expression. By screening potential miR-122 targets among TLR4, we found that there was a putative miR-122 target in TLR4 3′UTR. Mutations in the nt1603-nt1609 region of TLR4 3′UTR abandoned the impact of miR-122 on TLR4 expression. Over-expression/down-expression of miR-122 could influence the proliferation and the expression of natural immune factors.

**Conclusions:**

MiR-122 might target TLR4 and regulate host innate immunity in hepatoma cells, which revealed a new molecular mechanism of miR-122 on the regulation of innate immunity.

## Background

Hepatocellular carcinoma (HCC) is considered to be one of the most common malignancies in the world, which influences our health [1]. Up to now, there is not effective therapy strategy for HCC [2] because the pathogenic mechanism of HCC is highly complicated [3]. The tumorigenesis of HCC involves multistage processes, and it has been reported that abnormal Toll-like receptor (TLR) signaling pathways might contribute to the development and progression of HCC [4].

TLRs are mainly expressed in immune cells and play an important role in the homeostasis of the human immune system [5]. However, more evidence suggest that TLRs can also be expressed in many kinds of tumor cells, and the signaling pathways of TLRs can be associated with the progression of HCC through affecting the proliferation of tumor cells, evasion of immune surveillance and drug resistant [6–9]. For example, TLR4 can promote tumor development in human head and neck squamous cell carcinoma [10]. TLR4 signaling pathway could also induce COX-2/PGE2/STAT3 positive feedback loop to regulate the proliferation and drug resistance of HCC [11]. Recent research demonstrated that high expression of TLR4 was associated with microvascular invasion in HCC [12]. These observations indicate TLR4 displays critical roles in HCC progression.

MiR-122 is the most abundant miRNA in hepatocytes, which can be involved in hepatocyte proliferation, apoptosis and related with HCC [13–15]. It could directly target cyclin G1 [16], anti-apoptosis gene Bcl-w [17], WNT1 gene in WNT signaling pathway [18], etc. The decreasing effect of miR-122 in a subset of HCC characterized by poor prognosis was reported [19]. MiR-122 could also regulate IFN expression, which suggested that miR-122 might have a novel function and therapeutic application in hepatocarcinogenesis [20].

In this study, we examined the associations between miR-122 and TLR4 in HCC. Our data suggest that miR-122 modulates host’s innate immunity through blocking TLR4 and may play important role in the carcinogenesis in the liver.

## Methods

### MiR-122 expression in HCC samples from TCGA database

In this study, we examined miR-122 expression in HCC tissues by searching TCGA database (https://cancergenome.nih.gov/). We obtained the miRNA profiles of 372 HCC tissues and 49 control tissues. Afterward, miR-122 expression was examined from the miRNA profiles.

### Cell culture

Human hepatocellular carcinoma cell lines HepG2, Huh7, human normal LO2 cells and 293 T (Harbin Medical University) were cultured in Dulbecco’s modified Eagle’s medium (DMEM) supplemented with 10% fetal bovine serum (FBS) (Gibco, USA). Cell were incubated at 37 °C in a humidified atmosphere with 5% CO_2_.

### MiRNA target prediction

The potential targets of miR-122 in the TLR4 3′UTR were predicted by TargetScan- Human 7.1 (targetscan.org/vert_71) and miRanda (microrna.org/microrna/home.do) based on the complementary sequences.

### Nucleotides, plasmids, and LPS

The sense and anti-sense miR-122 mimic (5′-TGGAGTGTGACAATGGTGTTTG-3′, 5′-CAAACACCAUUGUCACACUCCA-3′), the 2′-O-methylated, anti-miR-122 oligo- nucleotide (AMO-122) (5′-CAAACACCAUUGUCACACUCCA-3′), and the negative controls were synthesized (GenePharma, China). The pmiR-RB-REPORT™-TLR4 (RIBOBIO, China) and Lipopolysaccharides (Sigma, USA) were obtained respectively.

### Cell treatment and transfection

HepG2, Huh7 and LO2 cell lines were seeded for 24 h, and transiently transfected with nucleotides/plasmids according to the instructions of Lipofectamine 2000 (Invitrogen, USA), followed by treatment with 10 μg/mL LPS (Sigma, USA). Cells were used for RNA and protein extractions 48 h post-transfection.

### RNA isolation and quantitative real-time PCR

Total RNA was extracted from HepG2, Huh7 and LO2 cells using TRIzol reagent (Invitrogen, USA), followed by reverse transcription. Quantitative real-time PCR (qPCR) was performed with SYBR PrimeScript Ex Taq (TaKaRa, Japan) according to manufacturer’s instructions. Relative expression levels were determined and were normalized to that of glyceraldehyde 3-phosphate dehydrogenase (GAPDH) and U6. Three duplicate wells were set for all reactions. The relative expression was calculated based on the formula 2 (−ΔΔCt). ΔΔCt values are ΔCt exp. - ΔCt cont. The primers used were showed as followed (Table 1).

**Table 1:** Sequences of real-time PCR primers used in this study

Gene	Primer Sequences(5′-3′)
TLR4	F: 5′-TTGAGCAGGTCTAGGGTGATTGAAC-3′
	R: 5′-ATGCGGACACACACACTTTCAAATA-3′
GAPDH	F: 5′-ATCACTGCCACCCAGAAGAC-3′
	R: 5′-TTTCTAGACGGCAGGTCAGG-3′
miR-122	RT: 5′-GTCGTATCCAGTGCGTGTCGTGGAGTCGGCAATTGC ACTGGATACGACCAAACA-3′
	F: 5′-GGGTGG AGTGTGACA ATGG-3′
	R: 5′-TGCGTGTCGTGGAGTC-3′
U6	RT: 5′-CGCTTCACGAATTTGCGTGTCAT-3′
	F: 5′-GCTTCGGCAGCACATATACTAAAAT-3′
	R: 5′-CGCTTCACGAATTTGCGTGTCAT-3′

### Western blot analysis

Total proteins were extracted by Pierce RIPA Buffer (Thermo) and separated by 12% SDS- polyacrylamide gel electrophoresis. A polyclonal TLR4 antibody (Cell Signaling Technology, USA) and the polyclonal antibody against Actin (Zhongshan Golden Bridge, China) were used for immunoblotting detection. The blots were stained with a SuperSignal kit (Pierce, Rockford, IL) and photographed by LAS4000 (Fujifilm, Japan). The ratio of TLR4 to Actin was used as a quantitative measurement of TLR4 regulation by miR-122.

### MTT

Cell proliferation was identified using the 3-(4,5-dimethylthiazol-2-yl)-2,5-diphenyl tetrazolium.

bromide assay (MTT). Cell proliferation was evaluated at 24, 48 and 72 h after transfection. The absorbance of the samples was determined at 490 nm. Three independent repeated experiments were performed.

### Plasmids construction

Genomic DNA was extracted in 293 T cells as a template, and TLR4 3′UTR gene fragment was amplified. According to the predicted binding of miR-122 and TLR4 3′ UTR, mutant types of TLR4 3′UTR were designed: TLR4 3′UTR-m1. The primers included primer wild (F: 5′-GGCG GCTCGAGGTTCATCCAGCCTCCTCAG-3′, R: 5′- AATGCGGCCGCCTCATTTCTCCCTTC CTCC-3′), primer mutant (F: 5′-AAATATT TTGTGAGGCATGTTCATTGTGGCACT-3′, R: 5′-TGAACATGCCTCACAAAATATTTTCTTGAAATT-3′). The TLR4 3′ UTR/TLR4 3′UTR- m1 transcript was cloned into the *Xho*I/*NotI* sites in the pmiR-RB-REPORT™ plasmid. For mutation analysis, we substituted the 7-nt core seed-matched site (ACACTCC) with complementary bases (TGTGAGG). The mutations in these plasmids were verified by DNA sequencing.

### Reporter assay

293 T cells were co-transfected with miR-122/miRNA-control and TLR4-WT plasmid/TLR4- MUT plasmid using Lipofectamine 2000. After 48 h, the cells were collected for application in the reporter system (Promega, USA) following the manufacturer’s instructions. All dual- luciferase reporter assays were carried out in triplicate within each experiment, and three independent experiments were conducted.

### Elisa

The productions of IL-6 and TNF-α in culture supernatant were measured using the Human IL-6 and TNF-α ELISA kits (Mlbio, China) according to the manufacturer’s instructions.

### Statistical analysis

Measurement data were represented as the mean ± standard error of the mean (SEM). Statistical analysis was performed using SigmaStat 3.0 (Systat Software, USA). The Student’s t test was used to evaluate the differences between two groups. When *P* < 0.05, the difference was considered to have statistical significance.

## Results

### Expression of miR-122 in HCC from TCGA database

**Fig. 1:**
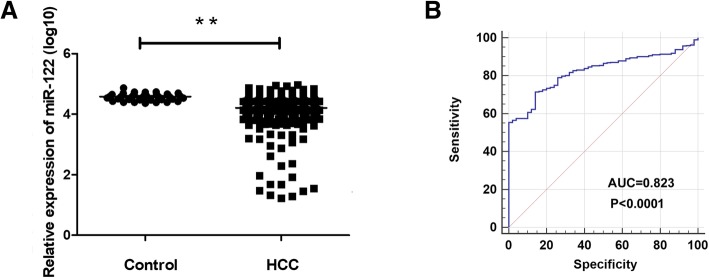
MiR-122 expression in TCGA samples and the diagnostic value. **a** The expression of miR-122 in 372 HCC and 49 control tissues. **b** The ROC curve was generated to assess the diagnostic ability of miR-122 in HCC and control tissues. The AUC was 0.823 (*p* < 0.001). (AUC = 0.823, *p* < 0.0001)

A total of 372 HCC cases and 49 adjacent control cases were collected from TCGA database. MiR-122 expression was decreased in the HCC group in comparison with the control group (*p* < 0.01) (Fig. 1a). The ROC curve assessed the diagnostic ability of miR-122 (AUC = 0.823, *p* < 0.0001) (Fig. 1b).

### The expression of miR-122 is downregulated and TLR4 was upregulated in hepatocytes

**Fig. 2:**
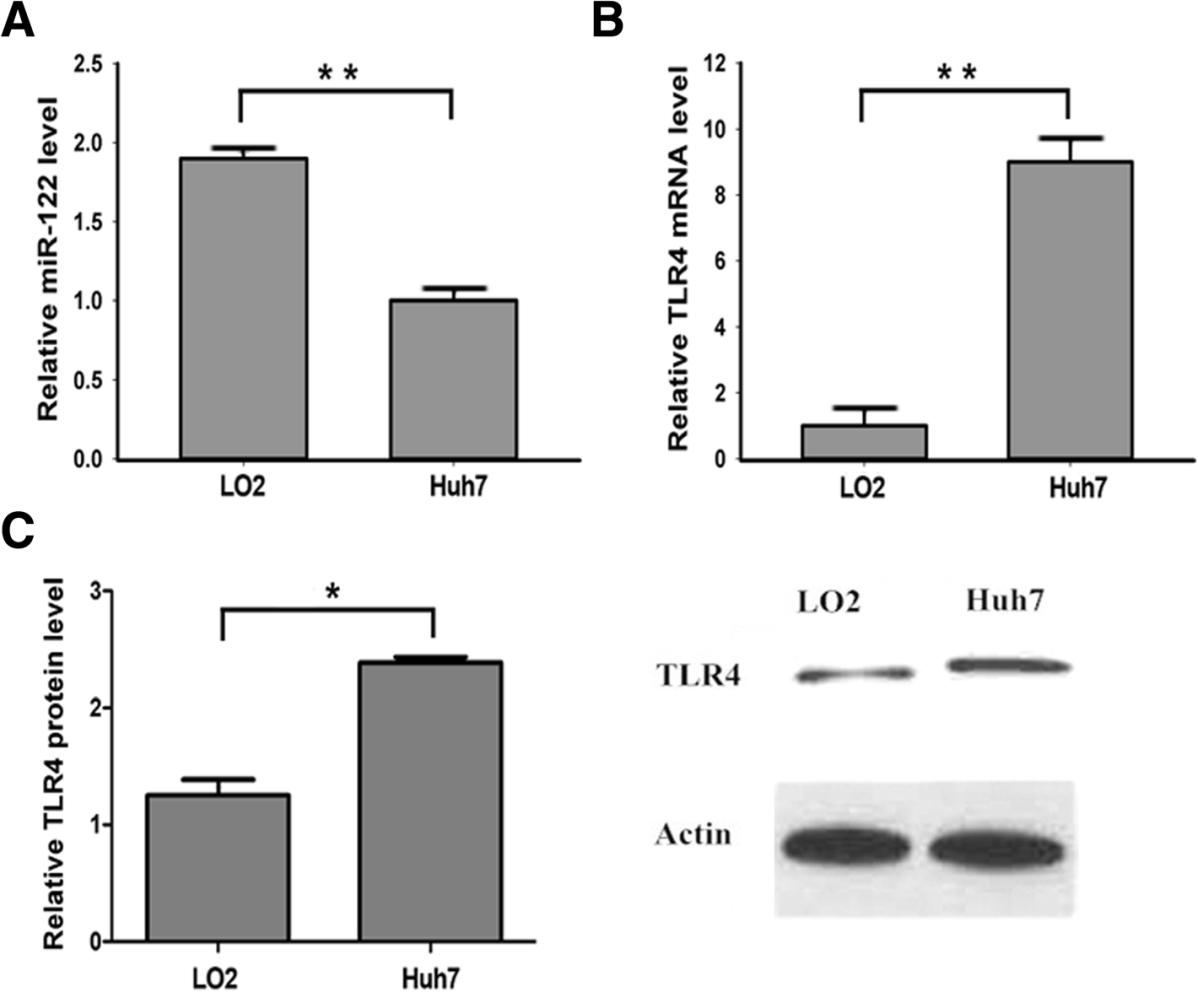
The expression of miR-122 is downregulated and TLR4 was upregulated in hepatocytes. **a** and **b** qPCR analysis of the relative miR-122 and TLR4 mRNA levels in hepatoma cells Huh7 compared with the normal human hepatocyte LO2 cells. **c** Western blot was used to measure the expression of TLR4, and beta-actin was used as an internal control. Values are presented as the mean ± SEM (*n* = 3). *P < 0.05, **P < 0.01

Previous studies showed that miR-122 downregulated in HCC tissue, while TLR4 levels varied between specimens. These findings imply that expression of miR-122 and TLR4 is inversely related in HCC. To ascertain the relationship between miR-122 and TLR4 in hepatocytes, we first compared their expression levels in human HCC cell lines Huh7 and human normal liver cells LO2. The results showed that miR-122 was over-expressed in LO2 cells in comparison with HCC Huh7 cells (Fig. 2a), which indicated that miR-122 might display a crucial role in hepatoma development. We next examined the TLR4 mRNA and protein expression by qPCR and western blot. The expression of TLR4 was found to be obviously up-regulated in HCC Huh7 cells compared with the LO2 cells, which was opposite to the expression level of miR-122 (Fig. 2b and c). This further suggested that TLR4 might have the negative correlation with miR-122 abundances in hepatocytes.

### MiR-122 could suppress the TLR4 expression

**Fig. 3:**
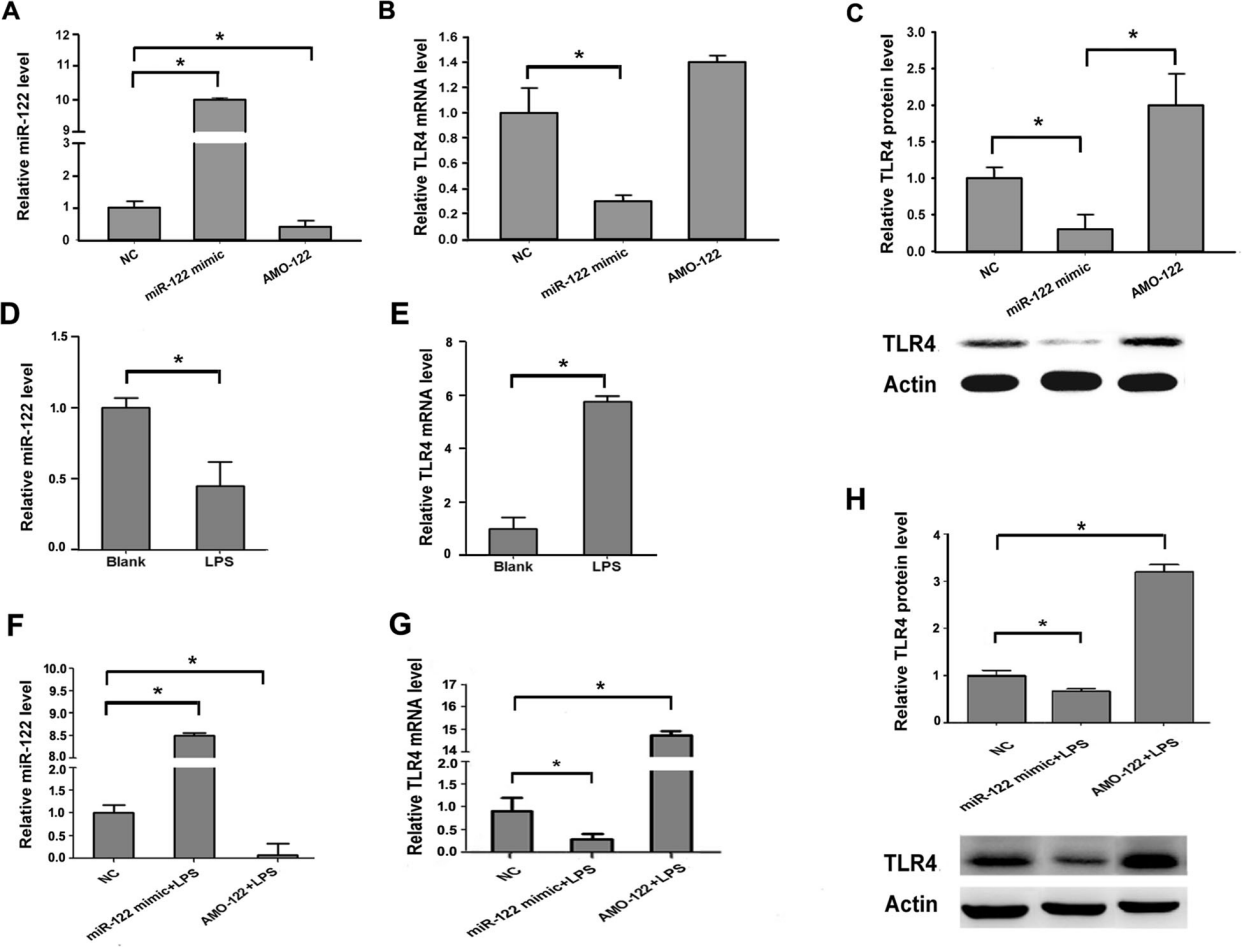
MiR-122 suppresses the TLR4 expression. **a** The expression of miR-122 in HepG2 cells transfected with miR-122 mimic and AMO-122 for 48 h. **b** and **c** The expression of TLR4 mRNA and protein. **d** and **e** The expressions of miR-122 and TLR4 mRNA in LO2 cells treated with LPS. **f**, **g** and **h** The expressions of miR-122, TLR4 mRNA and protein in LO2 cells transfected with miR-122 mimic/AMO-122, followed by LPS treatment. Values are presented as the mean ± SEM (*n* = 3). * refers to *P* < 0.05

To verify whether the expression of TLR4 could be regulated by miR-122, HepG2 cells were transfected with miR-122 mimic/AMO-122 and negative controls (NC) to determine the mRNA and protein expression levels of TLR4 by qPCR and western blotting, respectively. The level of miR-122 expression was examined first after transfection (Fig. 3a), which suggested that miR-122 mimic or AMO-122 could increase or reduce the expression of miR-122 and could be used in the following experiments. The mRNA and protein level of TLR4 was significantly downregulated in the miR-122-overexpressing HepG2 cells compared with the NC-transfected cells (*P* < 0.05) (Fig. 3b and c). These findings demonstrated that miR-122 may inhibit TLR4 expression in hepatocytes.

LPS can stimulate TLR4, leading to the high expression of TLR4 after LPS treatment, so the expressions of TLR4 and miR-122 were detected to identify the relationship between them. The results showed that miR-122 was down-regulated and TLR4 was up-regulated (Fig. 3d and e). To validate the influence of miR-122 on TLR4 expression in hepatocytes, miR-122 mimic was transfected to LO2 cells to reverse the decrease of miR-122, followed by LPS stimulation. The expression of miR-122 increased/decreased with miR-122 mimic/AMO-122 transfection after LPS treatment (Fig. 3f), and TLR4 expression was opposite to that of miR-122 (Fig. 3g and h), which were consistent with that of miR-122 mimic and AMO-122 transfection (Fig. 3b and c). These results confirm that miR-122 specifically suppresses the TLR4 expression.

### Prediction and confirmation of miR-122 targets in the TLR4 3′UTR

**Fig. 4:**
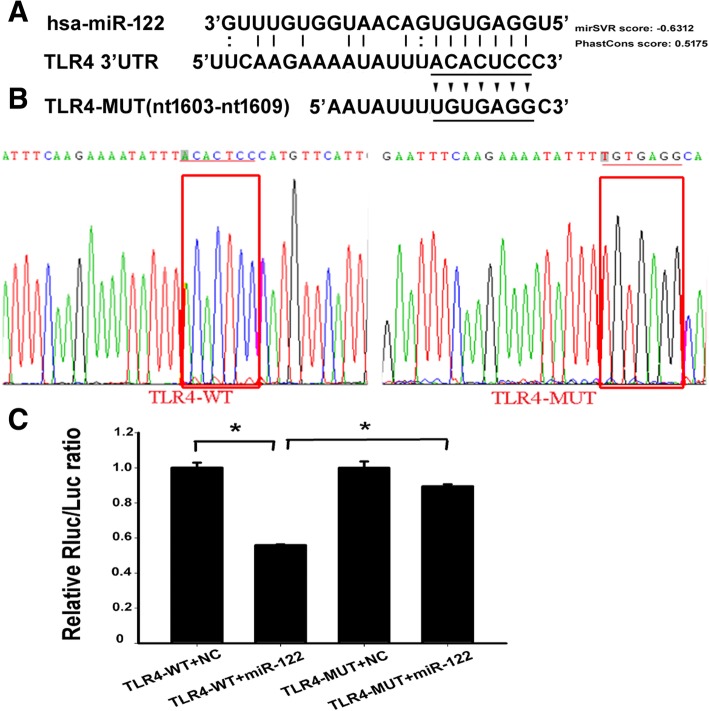
MiR-122 directly binds to the 3′UTR of TLR4 transcript. **a** Prediction of miR-122 targets in TLR4 3′UTR. The complementary sequences between miR-122 and TLR4 3′UTR (GenBank accession: NM_138554.4). **b** The mutated nucleotides in the putative targets of TLR4 and the sequencing data of the mutated pTLR4-MUT. The pTLR4-MUT were generated to minimize the match of miR-122 with TLR4 3′UTR in the region of nt1603-nt1609. The red boxes are the mutated regions. **c** MiR-122 mimic was co-transfected into 293 T cells with TLR4-WT or TLR4-MUT plasmid, respectively. TLR4 expression in the treated cells was observed at 48 h post-transfection. Values are presented as the mean ± SEM (*n* = 4). * refers to *P* < 0.05, ** refers to *P* < 0.01

To verify our hypothesis, we used the Targetscan and miRanda, web-based miRNA analysis tools, to screen potential miR-122 targets in TLR4 3′UTR. Interestingly, the analysis results indicated that there were 13 putative miR-122 binding sites located in the 3′UTR of TLR4 transcript and the target sequence was perfectly matched to the seed sequence of miR-122, which located in nt1603-nt1609 of TLR4 3′UTR (Fig. 4a). To clarify the interaction between miR-122 and TLR4 3′UTR, sequences corresponding to miR-122 seed binding sites were constructed. pmiR-RB-REPORT™ plasmids containing wild and mutated TLR4 3′UTR (TLR4-WT and TLR4-MUT) were generated by PCR. The TLR4-MUT plasmid contained mismatches in the common miR-122 seed region, and seven nucleotides between nt1603 and nt1609 were substituted from ACACTCC to TGTGAGG (Fig. 4b). Mutations in this region should block miR-122 binding and abrogate the miR-122-mediated repression of TLR4 expression. Using the same protocol above, miR-122 mimic and negative control were separately co-transfected into 293 T cells with TLR4-WT or TLR4-MUT. The TLR4 expression was measured by luciferase assay. Cells containing the TLR4-WT showed a significant and repeatable decrease in activity in the presence of mimics of miR-122 compared with the negative control (Fig. 4c). This suggested that TLR4 may be a miR-122 target. In contrast, the TLR4-MUT was co-transfected into 293 T cells with miR-122 mimic or a negative control. The expression of TLR4 did not change significantly upon treatment with mimics of miR-122 (Fig. 4c). These results demonstrated that miR-122 downregulated TLR4 expression via direct binding to site 1603–1609 in the 3′UTR of TLR4.

### MiR-122 negatively regulates the proliferation of HCC cells and production of Proinflammatory cytokines

**Fig. 5:**
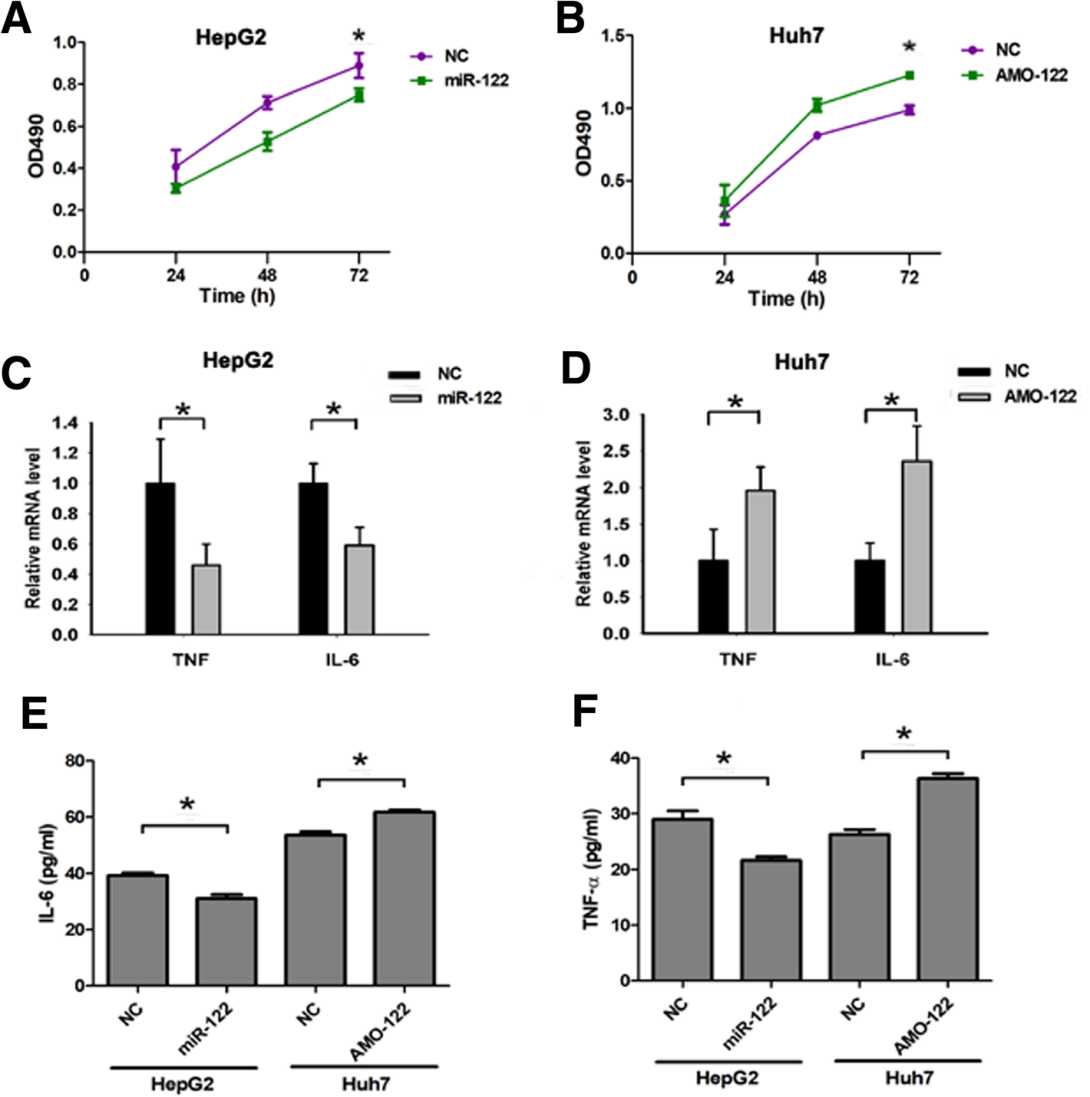
MiR-122 suppresses the proliferation and production of proinflammatory cytokines. **a** The proliferation of HepG2 cells was detected by MTT after transfecting with miR-122 mimic. **b** The proliferation of Huh7 cells was detected by MTT after transfecting with AMO-122. **c** The expression of TNF-α and IL-6 in HepG2 cells transfected with miR-122 mimic for 48 h. **d** The expression of TNF-α and IL-6 in Huh7 cells transfected with AMO-122 for 48 h. **e**, **f** The expression of IL-6 and TNF-α in HepG2/Huh7 cells transfected with miR-122 mimic/AMO- 122. Values are presented as the mean ± SEM (*n* = 3). *P < 0.05, **P < 0.01

To analyze whether miR-122 expression could regulate HCC cells proliferation, we transfected miR-122 mimic into HepG2 cells and AMO-122 into Huh7 cells. MTT results showed that increasing of miR-122 inhibited HepG2 cells proliferation, however, decreasing of miR-122 promoted Huh7 cells proliferation (Fig. 5a and b). To assess whether miR-122 may be involved in the regulation of innate response in the pathways, we also analyzed the expression of proinflammatory cytokines. MiR-122 overexpression/inhibition significantly inhibited/increased TNF-α and IL-6 production (Fig. 5a and b). These results indicated that miR-122 might act as a negative regulator of proliferation and TLR-triggered innate inflammatory response by suppressing the production of proinflammatory cytokines such as IL-6 and TNF-α.

## Discussion

TLR4 was functionally expressed on HCC cells, and many liver cells (Kupffer cells, hepatocytes, activated stellate cells) constantly express TLR4 [8, 21–24]. The expression of TLR4 was significantly increased in HBV- and HCV-infected livers, as well as in HCC tissues [25–28]. TLR4 signaling pathway induced by LPS can be associated with the survival and proliferation of cancer cells in HCC [29, 30]. TLR4 activation subsequently activates the downstream signaling pathways, leading to the expression of proinflammatory cytokines [31]. Several inflammatory mediators, such as TNF-*α*, IL-6, and IL-10, might have an effect in the initiation and progression of cancer [32], so the inflammatory microenvironment can be relate to the tumorigenesis of liver [33]. IL-6 is one of the proinflammatory cytokines, which have a typical protumorigenic effect [32]. IL-6 can activate signal transducer and activator of transcription 3 (STAT3) and upregulate the transcription of SOCS3 [34]. The activation of JAK/STAT3 pathways might be involved in HCC progression [35]. Notably, the upregulation of IL-6 in human HCC was shown to induce tumor development [36–38]. Tumor necrosis factor (TNF-α) is also one important inflammatory mediator that has been implicated in carcinogenesis [32, 39].

MiR-122, a liver-specific miRNA in human, is decreased in HCC tissues and plays a pivotal role in regulating the metabolism and hepatocarcinogenesis [40–44]. Silencing miR-122 can also induce the dedifferentiation of hepatocytes [45]. MiR-122 might be an intrinsic tumor suppressor gene [44, 46–48] and have an anti-inflammatory role in HCC [42]. Some results showed that some exosome-transported miRNAs can inhibit HCC growth in SCID mice [23, 49, 50], which might be one of the modes of the effect of miR-122. Furthermore, IL-6 and TNF-α can markedly decrease the expression level of miR-122 [51]. All these revealed that miR-122 may have therapeutic potential for preventing the progression of liver diseases [52].

The results of our study showed that miR-122 was downregulated and TLR4 was upregulated in HCC cell lines, which was consistent with the results in many reports. All these demonstrated a negative regulatory relationship between miR-122 and TLR4 in hepatocellular cell lines. These results are consistent with the study which transient transfected miR-122 mimic into HepG2 cells caused a decrease in TLR4 protein level. MiR-122 can also suppress the TLR4 expression with LPS stimulation in LO2 cells. We confirmed that miR-122 directly bound to the 3′-UTR of the TLR4 transcript and further suppress the translation of TLR4 by a luciferase reporter assay. In addition, we also examined the expression of IL-6 and TNF-α. As a TLR4 common downstream cytokine, the expression of IL-6 and TNF-α were also significantly down-regulated after intervention with mimics. Accordingly, we believe that miR-122 attenuates TLR4 signaling pathway via targeting TLR4 directly.

## Conclusions

In conclusion, our results indicated that miR-122 repressed the expression of TLR4 by binding to the 3′UTR of TLR4. Downregulating the expression of miR-122 could promote the expression of TLR4. The upregulation of miR-122 could regulate host innate immunity by TLR4 in hepatoma cells, which will provide a strategy for cancer treatment.

## Data Availability

The datasets generated and analyzed during the current study are available from the corresponding author on reasonable request.

## Abbreviations

AMO-122: Anti-miR-122 oligonucleotide
GAPDH: Glyceraldehyde-3-phosphate dehydrogenase
HCC: Hepatocellular carcinoma
IL: Interleukin
qRT-PCR: Quantitative RT-PCR
ROC: Receiver operation characteristic
SEM: Standard error of the mean
TLR: Toll-like receptor
TNF: Tumor necrosis factor
